# Thyroid Functioning and Fatigue in Women With Functional Somatic Syndromes – Role of Early Life Adversity

**DOI:** 10.3389/fphys.2018.00564

**Published:** 2018-05-23

**Authors:** Susanne Fischer, Charlotte Markert, Jana Strahler, Johanna M. Doerr, Nadine Skoluda, Mattes Kappert, Urs M. Nater

**Affiliations:** ^1^Clinical Psychology and Psychotherapy, Institute of Psychology, University of Zurich, Zurich, Switzerland; ^2^Clinical Biopsychology, Department of Psychology, University of Marburg, Marburg, Germany; ^3^Department of Psychotherapy and Systems Neuroscience, Faculty of Psychology and Sport Science, Justus Liebig University Giessen, Giessen, Germany; ^4^Clinical Psychology, Department of Psychology, University of Vienna, Vienna, Austria

**Keywords:** early life adversity, fatigue, fibromyalgia, functional somatic syndromes, hypothalamic-pituitary-thyroid axis, irritable bowel syndrome, stress

## Abstract

**Objective:** Fatigue is a core feature of functional somatic syndromes (FSS). Fatigue is also prominent in patients with thyroid diseases, which is unsurprising given the role of the hypothalamic-pituitary-thyroid (HPT) axis in regulating physiological energy demands. Research in healthy women has shown that early life adversity is linked with alterations in the HPT axis. In view of the substantial prevalence of early life adversity in patients with FSS, our aim was to investigate whether HPT functioning is related to (a) fatigue, and (b) early life adversity in these patients.

**Methods:** *N* = 33 female patients with FSS and *n* = 30 age-matched controls were recruited. Fasting morning blood samples were taken to determine thyroid-stimulating hormone (TSH), free triiodothyronine (fT3), and thyroxine (fT4). General, physical, and mental fatigue were measured via the multidimensional fatigue inventory (MFI). Early life adversity was measured using the childhood trauma questionnaire (CTQ).

**Results:** Patients with FSS did not differ from controls in any thyroid parameters (all *p* > 0.672). However, the lower the patients’ TSH and the higher their fT4, the greater was their general (β = -0.32, *p* = 0.064; β = 0.35, *p* = 0.038) and physical (β = -0.47, *p* = 0.007; β = 0.32, *p* = 0.077) fatigue. In addition, emotional neglect (β = -0.32, *p* = 0.057), physical neglect (β = -0.60, *p* = 0.001), physical abuse (β = -0.47, *p* = 0.015), and sexual abuse (β = -0.40, *p* = 0.026) were linked with lower TSH.

**Conclusion:** The lower TSH and the higher fT4, the more fatigue was reported by patients with FSS. In addition, lower TSH was linked with more early life adversity. Larger, prospective studies are warranted to determine whether HPT functioning may be a mediating pathway between early life adversity and fatigue in FSS.

## Introduction

Fatigue is a multidimensional phenomenon, which can be described on a physical and mental level. While physical fatigue refers to a state of bodily exhaustion, mental fatigue is characterized by reduced alertness (Smets et al., 1995). As a symptom, fatigue is present in several medical diseases, such as cancer (Minton et al., 2013). However, around a third of patients seen in primary care report medically unexplained fatigue, that is, fatigue in the absence of such conditions (Steinbrecher et al., 2011). In line with this, fatigue features prominently in several of the so-called functional somatic syndromes (FSS), which are characterized by different constellations of physical symptoms that are not attributable to any somatic disease (Wessely et al., 1999). For instance, fatigue is the core symptom of chronic fatigue syndrome (Fukuda et al., 1994), is a case-defining symptom of fibromyalgia syndrome (Wolfe et al., 2010), and is one of the most frequently reported and most distressing symptoms in patients with irritable bowel syndrome, as recently confirmed by meta-analysis (Han and Yang, 2016).

Fatigue is also one of the most frequent complaints in overt thyroid diseases, such as Hashimoto’s thyroiditis (Chaker et al., 2017) or Graves’ disease (De Leo et al., 2016). This is unsurprising, given that the hypothalamic-pituitary-thyroid (HPT) axis is involved in governing physiological energy demands (Medici et al., 2015), and thyroid hormone receptors are expressed in numerous bodily tissues, including the brain (Bauer et al., 2008). Accordingly, the HPT axis is a highly interesting endocrine system to investigate in patients with FSS. However, to date, only a handful of reports have been published on this issue. In chronic fatigue syndrome, findings from case-control studies are equivocal, with null findings (Allain et al., 1997) reported alongside results of elevated thyroid-stimulating hormone (TSH) (Moorkens et al., 2000), decreased free triiodothyronine (fT3) (Ruiz-Nunez et al., 2018), and decreased free thyroxine (fT4) (Fuite et al., 2008) in patients. One study examining patients with fibromyalgia syndrome observed lowered fT3 when compared to healthy controls (Riedel et al., 1998). This finding is contradicted by other studies, which failed to detect any differences in HPT parameters between patients and controls (Neeck and Riedel, 1992; Inal et al., 2014). When taken together, there is preliminary evidence that patients with FSS are affected by increased TSH and/or lowered circulating levels of thyroid hormones. However, research attempting to unravel the origins of these alterations is sparse.

Interestingly, there is evidence to suggest that early life adversity can modulate HPT functioning. For instance, a recent study found higher TSH in pregnant women with versus without childhood trauma (Moog et al., 2017). Similarly, altered circulating levels of fT3 were shown in women with childhood sexual abuse when compared to non-traumatized women, the direction of which depended on whether they had a menstrually related mood disorder or not (Bunevicius et al., 2012). Given the high prevalence of early life adversity in patients with FSS (Nater et al., 2011; Afari et al., 2014), one may thus hypothesize that only a subgroup of patients reporting such experiences is affected by alterations in the HPT axis. The aims of the present study were therefore (1) to test whether patients with FSS differ from healthy controls in thyroid functioning, and (2) to investigate, for the first time, whether thyroid functioning is related to the experience of fatigue and early life adversity in patients. We expected to find significant associations of thyroid parameters with fatigue severity and with the degree of early life adversity. Due to the female preponderance in patients with FSS, and to limit any confounding effects of gonadal hormones on HPT measures, only women were included in the present study.

## Materials and Methods

### Participants

A total of *n* = 33 patients with FSS were recruited from the general population, from local primary and secondary care services, and via self-help groups. Inclusion criteria were age 18 years or above, fluency in the German language, and fulfillment of research diagnostic criteria for chronic fatigue syndrome, fibromyalgia syndrome, or irritable bowel syndrome. Chronic fatigue syndrome was diagnosed according to the Centers for Disease Control and Prevention (CDC) criteria (Fukuda et al., 1994), fibromyalgia syndrome was diagnosed according to the American College of Rheumatology (ACR) 2010 criteria (Wolfe et al., 2010), and irritable bowel syndrome was diagnosed according to the Rome III criteria (Longstreth et al., 2006). Exclusion criteria were pregnancy, lactation, and having any major physical disease, such as cancer, hepatic, hematological, neurological, or autoimmune diseases. The presence of overt thyroid diseases was excluded by means of a physical examination and laboratory testing (see below); i.e., all thyroid hormone levels had to be within the normal range. In addition, the following major mental disorders were excluded by means of a structured clinical interview (see below): current major depressive disorder, substance abuse or dependence within the past two years, any eating disorder within the past five years, and any lifetime psychotic or bipolar disorder. A total of *n* = 30 healthy age-matched controls were recruited from the general population using newspaper advertisements and flyers.

### Protocol

All participants were scheduled for an appointment at our laboratory between 8 and 10 am. First, participants were physically examined, and a thorough medical history was obtained in order to exclude any major physical diseases. For the same reason, a fasting blood sample was taken for a complete blood count with leukocyte differential, and to determine sodium, potassium, calcium, phosphate, chloride, aspartate transaminase, alanine transaminase, alkaline phosphatase, bilirubin, glucose, protein, creatinine, glomerular filtration rate, urea, IgG, IgA, IgM, C-reactive protein, rheumatoid factor, albumin, and anti-nuclear antibodies. The same sample was used to determine TSH, fT3, and fT4. Finally, the structured clinical interview for DSM-IV (SCID) was performed to confirm the absence of any major mental disorders. All study investigators were trained psychologists with extensive experience in conducting the SCID. The study protocol was approved by the local ethics committee (University of Marburg) and written informed consent in accordance with the Declaration of Helsinki was obtained from all participants.

### Psychosocial Measures

All sociodemographic information was collected by means of a questionnaire. Medical histories including current intake of medication were obtained via a standardized form. *Chronic fatigue syndrome* was assessed by means of three questionnaires and in line with a previously published algorithm that is based on the CDC criteria (Nater et al., 2009). In brief, the multidimensional fatigue inventory (MFI) (Smets et al., 1995) was used to measure fatigue severity, the CDC Symptom Inventory (Wagner et al., 2005) was used to collect information about ancillary symptoms of chronic fatigue syndrome, and the Medical Outcomes Study 36-item short-form health survey (Ware and Sherbourne, 1992) was administered to inquire about limitations in physical activities, social activities, usual role activities because of physical health problems, and usual role activities because of emotional problems. *Fibromyalgia syndrome* was diagnosed by means of the Fibromyalgia Symptom Scale (Wolfe et al., 2011), which consists of the widespread pain index (WPI) and the symptom severity (SS) scale and allows the determination of fibromyalgia syndrome according to the ACR 2010 case definition (Wolfe et al., 2010). A WPI ≥ 7 and SS ≥ 5 or a WPI 3-6 and SS ≥ 9 is indicative of a diagnosis. *Irritable bowel syndrome* was established based on the Irritable Bowel Syndrome Mode (Drossman and Dumitrascu, 2006). This scale contains all relevant symptoms of irritable bowel syndrome and is scored based on the Rome III criteria. The MFI (see above) was also was used to measure general *fatigue*, physical fatigue, and mental fatigue in both patients and controls. The childhood trauma questionnaire (CTQ) (Bernstein et al., 2003) was administered to measure *early life adversity*. It distinguishes five trauma domains: emotional neglect, physical neglect, emotional abuse, physical abuse, and sexual abuse.

### Biological Measures

A fasting blood sample was taken between 8 and 10 am and sent immediately to the biochemical laboratory of the University Hospital, University of Marburg. TSH was analyzed using a competitive chemiluminescence assay (Lumi-Phos530, Beckman, Krefeld). Free T3 and T4 were measured via a chemiluminescence assay (Lumi-Phos530, Beckman, Krefeld). The intra-assay variation was *<*5% for all parameters and the inter-assay variance was *<*5% for TSH and *<*6% for fT3 and fT4.

### Statistical Analyses

All variables deviating from a normal distribution were transformed via natural logarithms (biological measures) or analyzed by means of non-parametric tests (psychosocial measures). Univariate analyses of variance (ANOVAs) were computed to compare patients and controls in terms of circulating TSH, fT3, and fT4. Next, multiple linear regression analyses were conducted to (1) test the association between fatigue levels (predictor variable) and thyroid parameters (criterion variable), and (2) test whether early life adversity (predictor variable) was linked with thyroid parameters (criterion variable). Age, body mass index (BMI), smoking, and intake of medication were controlled for in all analyses. Descriptives are given as median and interquartile range (IQR) or frequencies. All analyses were conducted using the Statistical Package for the Social Sciences (SPSS), version 22, and the level of significance was set at 5%.

## Results

### Participant Characteristics

Characteristics of patients and controls are reported in **Table 1**. On average, the women in our sample were middle-aged, of normal weight, and around a fifth were smokers. Fourteen (42%) patients fulfilled criteria for both chronic fatigue syndrome and fibromyalgia syndrome, eight (24%) had chronic fatigue syndrome and irritable bowel syndrome, seven (21%) had fibromyalgia syndrome and irritable bowel syndrome, and six (18%) fulfilled criteria for all three syndromes. Patients and controls were statistically equal regarding sociodemographic and lifestyle variables, except for a marginally higher BMI in patients (trend). Patients had significantly higher levels of general, physical, and mental fatigue, and all scores were in the top quartile of the general population. Patients did not differ from healthy controls in terms of moderate to severe childhood trauma, with the exception that they tended to report more emotional abuse.

**Table 1:** Sociodemographic, lifestyle, and clinical characteristics of patients with functional somatic syndromes (FSS) and healthy controls.

	Patients (*n* = 33)	Controls (*n* = 30)	Statistics
Age (years)	43 (32)	32 (25)	*U* = -1.086, *p* = 0.278
Body mass index (BMI)	23 (4)	22 (3)	*U* = -1.707, *p* = 0.088
Smoking (yes)	7 (21%)	6 (20%)	χ^2^= 0.033, *p* = 0.856
**Functional somatic syndrome**			
Chronic fatigue syndrome	18 (55%)	0	n.a.
Fibromyalgia syndrome	17 (52%)	0	n.a.
Irritable bowel syndrome	20 (61%)	0	n.a.
**Medication**			
Analgesics	7 (21%)	0	n.a.
Antidepressants	3 (9%)	0	n.a.
Antihypertensives	5 (15%)	0	n.a.
**Fatigue (MFI)**			
General	14 (5)	6 (3)	*U* = -6.252, *p <* 0.001
Physical	12 (8)	5 (2)	*U* = -5.845, *p <* 0.001
Mental	11 (7)	5 (3)	*U* = -5.157, *p <* 0.001
**Early life adversity (CTQ)**			
Emotional neglect	10 (30%)	6 (20%)	χ^2^= 0.604, *p* = 0.437
Physical neglect	11 (33%)	7 (23%)	χ^2^= 0.493, *p* = 0.483
Emotional abuse	14 (42%)	6 (20%)	χ^2^= 3.348, *p* = 0.067
Physical abuse	4 (12%)	5 (17%)	χ^2^= 0.336, *p* = 0.721
Sexual abuse	6 (18%)	5 (17%)	χ^2^= 0.008, *p* = 0.929

*Descriptive statistics are given as median and interquartile range (IQR) or frequencies. Group comparisons were conducted using Mann–Whitney U-tests, Pearson’s chi-squared test, and Fisher’s exact test. CTQ, childhood trauma questionnaire (cut-offs for moderate to severe childhood trauma); MFI, multidimensional fatigue inventory (score range 5–20).*

### Thyroid Functioning in Patients Versus Controls

**FIGURE 1:**
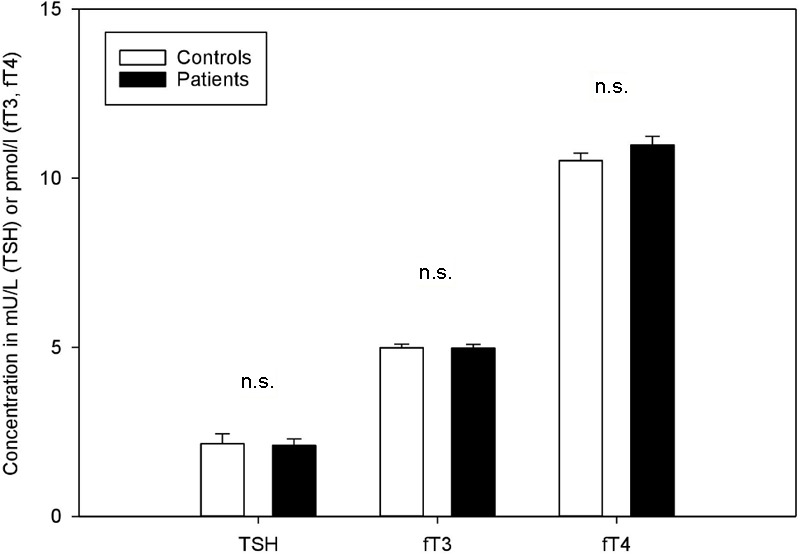
Thyroid parameters in patients with functional somatic syndromes (FSS) and healthy controls. Bars represent mean values and standard errors. Group comparisons did not yield any significant differences.

As is evident from **Figure 1**, patients with FSS and healthy controls did not differ in circulating TSH [2 (1.8) mU/L vs. 1.9 (1.5) mU/L; *F*(1,49) = 0.181, *p* = 0.672], fT3 [5 (0.9) pmol/l vs. 5.1 (1) pmol/l; *F*(1,49) = 0.037, *p* = 0.848], or fT4 [11 (2) pmol/l vs. 11 (1.4) pmol/l; *F*(1,49) = 0.071, *p* = 0.791].

### Thyroid Functioning and Fatigue

**FIGURE 2:**
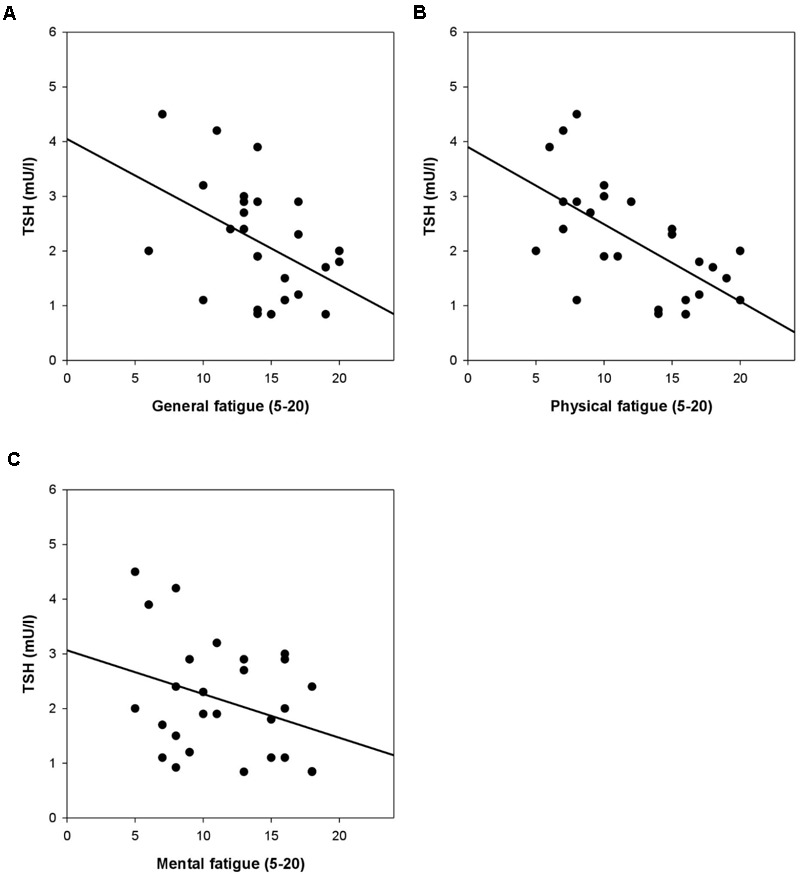
**(A–C)** Thyroid-stimulating hormone (TSH) and general, physical, and mental fatigue [multidimensional fatigue inventory (MFI)] in patients with FSS. Controlling for age, Body mass index (BMI), smoking, and intake of medication, the association between TSH and physical fatigue was significant (β = -0.47, *p* = 0.007), while there was a trend in terms of TSH and general fatigue (β = -0.32, *p* = 0.064). By contrast, TSH was found unrelated to mental fatigue (β = -0.29, *p* = 0.108).

There were marginally to highly significant relationships between circulating TSH and patients’ general (β = -0.32, *p* = 0.064) and physical fatigue levels (β = -0.47, *p* = 0.007), while mental fatigue was not linked with TSH (β = -0.29, *p* = 0.108; see also **Figures 2A–C**). Similar findings emerged in terms of fT4, which had significant links with general fatigue (β = 0.35, *p* = 0.038) and marginally significant links with physical fatigue (β = 0.32, *p* = 0.077), while mental fatigue was found unrelated to fT4 (β = 0.13, *p* = 0.481). By contrast, none of the regression models predicting fT3 (all *p* > 0.328) was significant.

There were no relationships between thyroid parameters and fatigue in healthy controls (all models *p* > 0.474).

### Early Life Adversity and Thyroid Functioning

**FIGURE 3:**
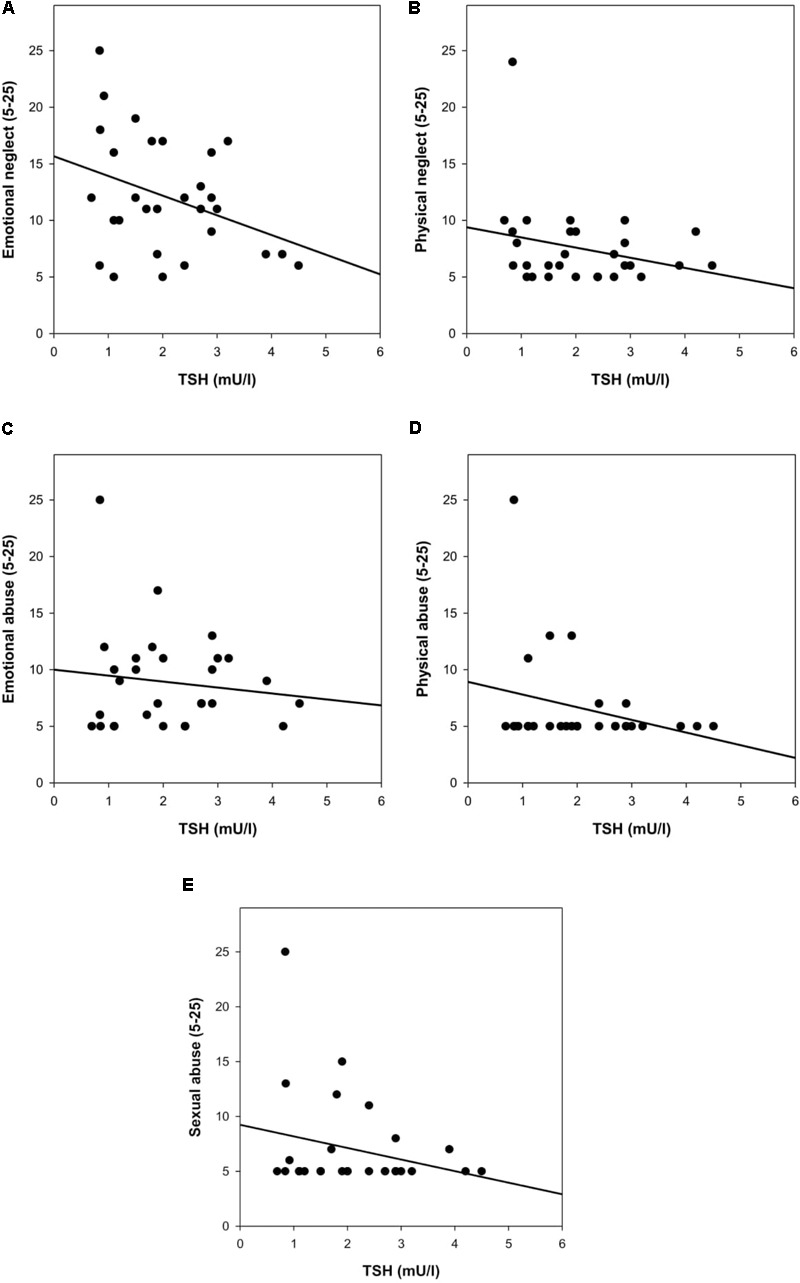
**(A–E)** Early life adversity [childhood trauma questionnaire (CTQ)] and TSH in patients with FSS. Controlling for age, Body mass index (BMI), smoking, and intake of medication, the association of physical neglect (β = -0.60, *p* = 0.001), physical abuse (β = -0.47, *p* = 0.019), and sexual abuse (β = -0.40, *p* = 0.030) with TSH was significant, and there was a trend in terms of emotional neglect (β = -0.33, *p* = 0.064). There was no significant association between emotional abuse and TSH (β = -0.31, *p* = 0.122).

In patients, nearly all forms of childhood trauma were marginally to highly significantly related to circulating levels of TSH (emotional neglect: β = -0.33, *p* = 0.064; physical neglect: β = -0.60, *p* = 0.001; emotional abuse: β = -0.31, *p* = 0.122; physical abuse: β = -0.47, *p* = 0.019; sexual abuse: β = -0.40, *p* = 0.030; see also **Figures 3A–E**). No such associations were observed for fT3 (all models *p* > 0.157) and the same was true for fT4, although there was a trend-level association with physical neglect (β = 0.31, *p* = 0.099).

There were no relationships between childhood trauma and thyroid parameters in healthy controls (all models *p* > 0.146).

## Discussion

The present study has two main findings: First, patients with FSS did not differ from their healthy counterparts in terms of thyroid functioning, but a lower TSH and higher fT4 concentration was associated with greater physical and general fatigue, respectively. Second, the more early life adversity patients experienced, the lower their current TSH levels.

The first finding is in line with previous studies which failed to find any differences in thyroid parameters between patients with FSS and healthy controls (Neeck and Riedel, 1992; Allain et al., 1997; Inal et al., 2014). However, others reported elevated TSH (Moorkens et al., 2000), lowered circulating fT3 (Riedel et al., 1998; Ruiz-Nunez et al., 2018), or attenuated fT4 (Fuite et al., 2008) in patients with chronic fatigue syndrome or fibromyalgia syndrome. These discrepancies are likely attributable to sample characteristics. For instance, different case definitions of chronic fatigue syndrome have been published, such as the Oxford (Sharpe et al., 1991) and CDC criteria (Fukuda et al., 1994), and there have been numerous modifications to the ACR criteria for fibromyalgia syndrome over the past two decades. In addition, ours was the first study to include patients with irritable bowel syndrome, which despite the syndrome overlap among FSS (Aaron and Buchwald, 2001; Fischer et al., 2013), naturally hampers any comparison with prior research. Similarly, we were careful to exclude patients with overt hypothyroidism or hyperthyroidism as determined by laboratory testing, while not all previous FSS studies adhered to such procedures. While the CDC criteria for chronic fatigue syndrome are very specific in describing conditions exclusionary to its diagnosis and how these should be established (Fukuda et al., 1994), the ACR criteria are far less restrictive by allowing any somatic disease to co-occur with fibromyalgia syndrome (Wolfe et al., 2010). It is thus possible that some of the previous studies contained cases with autoimmune thyroid diseases, which could have inflated any subtle differences between patients and healthy controls. Interestingly, we were still able to demonstrate a significant negative relationship between TSH concentrations and physical fatigue and a positive relationship between fT4 and general fatigue in the present sample. This either suggests that lower TSH and higher fT4 lead to fatigue in patients with FSS, or that prolonged periods of fatigue decrease TSH and increase fT4. Experimental studies may prove useful in examining these hypotheses in greater detail. In addition, the mechanisms underlying the observed links between TSH and physical fatigue should be explored in greater detail by assessing to what extent the thyroid’s secretory capacity or activity of peripheral deiodinases may play a role in this (see, e.g., Ruiz-Nunez et al., 2018).

The second finding of this study – a negative association between early life adversity and TSH levels in patients with FSS – is novel. Very few studies have investigated how HPT functioning in general may be altered as a result of adverse experiences during childhood. Most recently, Moog et al. (2017) were able to demonstrate that women with moderate to severe childhood trauma had a significantly enhanced risk of subclinical hypothyroidism during pregnancy, as indicated by higher circulating levels of TSH. Another study found altered fT3 in women reporting a history of sexual abuse when compared to non-abused women (Bunevicius et al., 2012). When taken together with the finding of the present study, it is tempting to assume that subtle alterations in HPT functioning represent a mediating pathway between early life adversity and fatigue as present in FSS. This would resonate well with the notion of thyroid allostasis, which describes the wear and tear of the HPT axis in response to various stressors (see Chatzitomaris et al., 2017 for a recent review). The early life period might be particularly sensitive to permanent stress-induced alterations of endocrine systems. Animal research has shown that lower maternal caregiving resulted in lower T3 and higher T4 levels, which appeared to be due to tactile stimulation (Hellstrom et al., 2012). This in turn was linked with epigenetic modifications within the gene encoding the glucocorticoid receptor, thus influencing hypothalamic-pituitary-adrenal (HPA) axis functioning. Given the extant evidence on HPA alterations in patients with FSS (Nater et al., 2011), the HPT axis could thus be a crucial player in translating early life adversity into FSS. Notably, certain types of childhood trauma, namely physical neglect, physical abuse, and sexual abuse appear to have a particularly strong impact on HPT functioning, as suggested by the data of this study and other research in patients with PTSD, borderline personality disorder, or depressive disorders (e.g., Friedman et al., 2005; Plaza et al., 2010; Sinai et al., 2014). This aligns well with recent evidence for a differential impact of trauma subtypes on the neural architecture (Heim et al., 2013). However, the mechanisms underlying these findings have not been examined yet. An alternative explanation is that the CTQ provides more valid estimates of physical abuse, physical neglect, and sexual abuse when compared to emotional abuse and neglect, which is indeed suggested by the original validation study (Bernstein et al., 2003). In conclusion, further studies with are required to directly test the outlined mediation hypotheses, and preferably in larger samples of patients, since extreme levels of abuse and neglect (as present in some individuals with FSS) may otherwise have a disproportionately high impact.

This study presents with a number of strengths, such as our careful selection of patients with FSS. All patients fulfilled criteria for the most widely used case definitions for each FSS and were free of any major physical and mental illnesses. In addition, this was the first study to investigate how one of the most frequent and most disabling symptoms of these patients – fatigue – is related to thyroid functioning. Finally, we provided initial evidence that early life adversity is linked with thyroid functioning in this group of individuals. A number of limitations equally deserve mention. First, the decision to exclude men from the study means that replication of our findings in a mixed sample is warranted. Second, our sample was not large enough for a meaningful stratification according to the presence or absence of early life adversity. We were, however, able to undertake correlation analyses, which may even be more informative given that early life adversity is not a dichotomous phenomenon. Third, prospective studies are required to answer the question of whether subtle alterations in the relationship between thyroid functioning and fatigue may ensue as a consequence of early life adversity, or whether such alterations precede adverse circumstances.

In sum, the present study provides initial evidence of a link between TSH and fT4 concentrations and fatigue as experienced by patients with FSS. Moreover, TSH levels were directly linked with early life adversity in patients. The HPT axis has been studied extensively in conditions which frequently co-occur with FSS (Henningsen et al., 2003; Fischer et al., 2013), such as anxiety disorders (Fischer and Ehlert, 2018) or major depression (Fountoulakis et al., 2006). In depression, this research has led to the development of effective therapies, such as the augmentation of antidepressant treatment with T3 in patients with treatment resistance. We would thus encourage future studies to continue this promising line of research in patients with FSS. We believe that larger, prospective studies in concert with experimental studies testing the integrity of the HPT axis in a standardized manner are most likely to advance the field at this stage.

## Author Contributions

UN conceived the study. SF, CM, MK, JS, JD, and NS implemented the study. SF drafted the article. UN, CM, JS, JD, NS, and MK revised the article for important intellectual content.

## Conflict of Interest Statement

The authors declare that the research was conducted in the absence of any commercial or financial relationships that could be construed as a potential conflict of interest.

## References

[B1] Aaron L. A. Buchwald D. (2001). A review of the evidence for overlap among unexplained clinical conditions. 134 868–881. 10.7326/0003-4819-134-9_Part_2-200105011-00011 11346323

[B2] Afari N. Ahumada S. M. Wright L. J. Mostoufi S. Golnari G. Reis V. (2014). Psychological trauma and functional somatic syndromes: a systematic review and meta-analysis. 76 2–11. 10.1097/PSY.0000000000000010 24336429 PMC3894419

[B3] Allain T. J. Bearn J. A. Coskeran P. Jones J. Checkley A. Butler J. (1997). Changes in growth hormone, insulin, insulin-like growth factors (IGFs), and IGF-binding protein-1 in chronic fatigue syndrome. 41 567–573. 10.1016/S0006-3223(96)00074-1 9046989

[B4] Bauer M. Goetz T. Glenn T. Whybrow P. C. (2008). The thyroid-brain interaction in thyroid disorders and mood disorders. 20 1101–1114. 10.1111/j.1365-2826.2008.01774.x 18673409

[B5] Bernstein D. P. Stein J. A. Newcomb M. D. Walker E. Pogge D. Ahluvalia T. (2003). Development and validation of a brief screening version of the Childhood Trauma Questionnaire. 27 169–190. 10.1016/S0145-2134(02)00541-0 12615092

[B6] Bunevicius A. Leserman J. Girdler S. S. (2012). Hypothalamic-pituitary-thyroid axis function in women with a menstrually related mood disorder: association with histories of sexual abuse. 74 810–816. 10.1097/PSY.0b013e31826c3397 23001392 PMC3465520

[B7] Chaker L. Bianco A. C. Jonklaas J. Peeters R. P. (2017). Hypothyroidism. 390 1550–1562. 10.1016/S0140-6736(17)30703-1 PMC6619426 28336049

[B8] Chatzitomaris A. Hoermann R. Midgley J. E. Hering S. Urban A. Dietrich B. (2017). Thyroid allostasis-adaptive responses of thyrotropic feedback control to conditions of strain, stress, and developmental programming. 8:163. 10.3389/fendo.2017.00163 28775711 PMC5517413

[B9] De Leo S. Lee S. Y. Braverman L. E. (2016). Hyperthyroidism. 388 906–918. 10.1016/S0140-6736(16)00278-6 PMC5014602 27038492

[B10] Drossman D. A. Dumitrascu D. L. (2006). Rome III: new standard for functional gastrointestinal disorders. 15 237–241. 17013448

[B11] Fischer S. Ehlert U. (2018). Hypothalamic-pituitary-thyroid (HPT) axis functioning in anxiety disorders. A systematic review. 35 98–110. 10.1002/da.22692 29064607

[B12] Fischer S. Gaab J. Ehlert U. Nater U. M. (2013). Prevalence, overlap, and predictors of functional somatic syndromes in a student sample. 20 184–193. 10.1007/s12529-012-9266-x 23055025

[B13] Fountoulakis K. N. Kantartzis S. Siamouli M. Panagiotidis P. Kaprinis S. Iacovides A. (2006). Peripheral thyroid dysfunction in depression. 7 131–137. 10.1080/15622970500474739 16861138

[B14] Friedman M. J. Wang S. Jalowiec J. E. McHugo G. J. McDonagh-Coyle A. (2005). Thyroid hormone alterations among women with posttraumatic stress disorder due to childhood sexual abuse. 57 1168–1192. 10.1016/j.biopsych.2005.01.019 15866559

[B15] Fuite J. Vernon S. D. Broderick G. (2008). Neuroendocrine and immune network re-modeling in chronic fatigue syndrome: an exploratory analysis. 92 393–399. 10.1016/j.ygeno.2008.08.008 18775774

[B16] Fukuda K. Straus S. E. Hickie I. Sharpe M. C. Dobbins J. G. Komaroff A. (1994). The chronic fatigue syndrome: a comprehensive approach to its definition and study. International Chronic Fatigue Syndrome Study Group. 121 953–959. 10.7326/0003-4819-121-12-199412150-00009 7978722

[B17] Han C. J. Yang G. S. (2016). Fatigue in irritable bowel syndrome: a systematic review and meta-analysis of pooled frequency and severity of fatigue. 10 1–10. 10.1016/j.anr.2016.01.003 27021828

[B18] Heim C. M. Mayberg H. S. Mletzko T. Nemeroff C. B. Pruessner J. C. (2013). Decreased cortical representation of gential somatosensory field after childhood sexual abuse. 170 616–623. 10.1176/appi.ajp.2013.12070950 23732967

[B19] Hellstrom I. C. Dhir S. K. Dirio J. C. Meaney M. J. (2012). Maternal licking regulates hippocampal glucocorticoid transcription through a thyroid-hormone-serotonin-NGFI-A signalling cascade. 367 2495–2510. 10.1098/rstb.2012.0223 22826348 PMC3405683

[B20] Henningsen P. Zimmermann T. Sattel H. (2003). Medically unexplained physical symptoms, anxiety, and depression: a meta-analytic review. 65 528–533. 10.1097/01.PSY.0000075977.90337.E7 12883101

[B21] Inal S. Inal E. E. Okyay G. U. Ozturk G. T. Onec K. Guz G. (2014). Fibromyalgia and nondipper circadian blood pressure variability. 20 422–426. 10.1097/RHU.0000000000000189 25417678

[B22] Longstreth G. F. Thompson W. G. Chey W. D. Houghton L. A. Mearin F. Spiller R. C. (2006). Functional bowel disorders. 130 1480–1491. 10.1053/j.gastro.2005.11.061 16678561

[B23] Medici M. Visser W. E. Visser T. J. Peeters R. P. (2015). Genetic determination of the hypothalamic-pituitary-thyroid axis: where do we stand? 36 214–244. 10.1210/er.2014-1081 25751422

[B24] Minton O. Berger A. Barsevick A. Cramp F. Goedendorp M. Mitchell S. A. (2013). Cancer-related fatigue and its impact on functioning. 119 2124–2130. 10.1002/cncr.28058 23695924

[B25] Moog N. K. Heim C. M. Entringer S. Kathmann N. Wadhwa P. D. Buss C. (2017). Childhood maltreatment is associated with increased risk of subclinical hypothyroidism in pregnancy. 84 190–196. 10.1016/j.psyneuen.2017.07.482 28755549 PMC5572821

[B26] Moorkens G. Berwaerts J. Wynants H. Abs R. (2000). Characterization of pituitary function with emphasis on GH secretion in the chronic fatigue syndrome. 53 99–106. 10.1046/j.1365-2265.2000.01049.x 10931086

[B27] Nater U. M. Fischer S. Ehlert U. (2011). Stress as a pathophysiological factor in functional somatic syndromes. 7 152–169. 10.2174/157340011796391184

[B28] Nater U. M. Lin J. M. Maloney E. M. Jones J. F. Tian H. Boneva R. S. (2009). Psychiatric comorbidity in persons with chronic fatigue syndrome identified from the Georgia population. 71 557–565. 10.1097/PSY.0b013e31819ea179 19414619

[B29] Neeck G. Riedel W. (1992). Thyroid function in patients with fibromyalgia syndrome. 19 1120–1122. 1512769

[B30] Plaza A. Garcia-Esteve L. Ascaso C. Navarro P. Gelabert E. Halperin I. (2010). Childhood sexual abuse and hypothalamic-pituitary-thyroid axis in postpartum depression. 122 159–163. 10.1016/j.jad.2009.07.021 19740549

[B31] Riedel W. Layka H. Neeck G. (1998). Secretory pattern of GH, TSH, thyroid hormones, ACTH, cortisol, FSH, and LH in patients with fibromyalgia syndrome following systemic injection of the relevant hypothalamic-releasing hormones. 57 81–87. 10.1007/s003930050242 10025090

[B32] Ruiz-Nunez B. Tarasse R. Vogelaar E. F. Jannecke Dijck-Brouwer D. A. Muskiet F. A. J. (2018). Higher prevalence of “low T3 syndrome” in chronic fatigue syndrome: a case-control study. 9:97 10.3389/fendo.2018.00097 PMC5869352 29615976

[B33] Sharpe M. C. Archard L. C. Banatvala J. E. Borysiewicz L. K. Clare A. W. David A. (1991). A report–chronic fatigue syndrome: guidelines for research. 84 118–121. 10.1177/014107689108400224 PMC1293107 1999813

[B34] Sinai C. Hirvikoski T. Nordström A. L. Nordström P. Nilsonne A. Wilczek A. (2014). Hypothalamic pituitary thyroid axis and exposure to interpersonal violence in childhood among women with borderline personality disorder. 16:5. 10.3402/ejpt.v5.23911 24959326 PMC4024607

[B35] Smets E. M. Garssen B. Bonke B. De Haes J. C. (1995). The Multidimensional Fatigue Inventory (MFI) psychometric qualities of an instrument to assess fatigue. 39 315–325. 10.1016/0022-3999(94)00125-O 7636775

[B36] Steinbrecher N. Koerber S. Frieser D. Hiller W. (2011). The prevalence of medically unexplained symptoms in primary care. 52 263–271. 10.1016/j.psym.2011.01.007 21565598

[B37] Wagner D. Nisenbaum R. Heim C. Jones J. F. Unger E. R. Reeves W. C. (2005). Psychometric properties of the CDC Symptom Inventory for assessment of chronic fatigue syndrome. 3:8. 10.1186/1478-7954-3-8 16042777 PMC1183246

[B38] Ware J. E. Jr. Sherbourne C. D. (1992). The MOS 36-item short-form health survey (SF-36). I. Conceptual framework and item selection. 30 473–483. 10.1097/00005650-199206000-00002 1593914

[B39] Wessely S. Nimnuan C. Sharpe M. (1999). Functional somatic syndromes: one or many? 354 936–939. 10.1016/S0140-6736(98)08320-2 10489969

[B40] Wolfe F. Clauw D. J. Fitzcharles M. A. Goldenberg D. L. Hauser W. Katz R. S. (2011). Fibromyalgia criteria and severity scales for clinical and epidemiological studies: a modification of the ACR preliminary diagnostic criteria for fibromyalgia. 38 1113–1122. 10.3899/jrheum.100594 21285161

[B41] Wolfe F. Clauw D. J. Fitzcharles M. A. Goldenberg D. L. Katz R. S. Mease P. (2010). The American College of Rheumatology preliminary diagnostic criteria for fibromyalgia and measurement of symptom severity. 62 600–610. 10.1002/acr.20140 20461783

